# Adaptive Digital Hologram Binarization Method Based on Local Thresholding, Block Division and Error Diffusion

**DOI:** 10.3390/jimaging8020015

**Published:** 2022-01-18

**Authors:** Pavel A. Cheremkhin, Ekaterina A. Kurbatova, Nikolay N. Evtikhiev, Vitaly V. Krasnov, Vladislav G. Rodin, Rostislav S. Starikov

**Affiliations:** Laser Physics Department, Institute for Laser and Plasma Technologies, National Research Nuclear University MEPhI (Moscow Engineering Physics Institute), Kashirskoe Shosse 31, 115409 Moscow, Russia; velana1511@mail.ru (E.A.K.); evtikhiev@mail.ru (N.N.E.); vitaly.krasnov@mail.ru (V.V.K.); holo_mephi@mail.ru (V.G.R.); rstarikov@mail.ru (R.S.S.)

**Keywords:** holography, digital holography, optical information processing, digital micromirror device, image binarization, optical image processing, 3D-display, error diffusion, computer-generated hologram, digital image processing

## Abstract

High-speed optical reconstruction of 3D-scenes can be achieved using digital holography with binary digital micromirror devices (DMD) or a ferroelectric spatial light modulator (fSLM). There are many algorithms for binarizing digital holograms. The most common are methods based on global and local thresholding and error diffusion techniques. In addition, hologram binarization is used in optical encryption, data compression, beam shaping, 3D-displays, nanofabrication, materials characterization, etc. This paper proposes an adaptive binarization method based on a combination of local threshold processing, hologram division into blocks, and error diffusion procedure (the LDE method). The method is applied for binarization of optically recorded and computer-generated digital holograms of flat objects and three-dimensional scenes. The quality of reconstructed images was compared with different methods of error diffusion and thresholding. Image reconstruction quality was up to 22% higher by various metrics than that one for standard binarization methods. The optical hologram reconstruction using DMD confirms the results of the numerical simulations.

## 1. Introduction

High-speed optical digital holographic reconstruction is one of the promising approaches for 3D-TV development [1,2,3,4] and for beam shaping [5]. This approach can be realized by using fast binary digital micromirror devices (DMD) with a frame rate even higher than 30 kHz [6]. Depending on the control signal of the micromirror, DMD reflects incident radiation in two directions. One direction corresponds to the light pixel position and the other to the dark pixel position. For grayscale images displayed using DMD, pulse width modulation can be used [7,8]. However, the frame rate of such system is significantly reduced [8]. In addition, quality of reconstruction usually does not exceed the case of the displayed binary holograms [9,10]. As a result, binary holograms are more useful for the majority of DMD applications. Another popular type of spatial light modulator is based on liquid crystals. The typical frame rate for liquid crystal modulators is significantly lower than that one for DMD [11,12] and equal to 60 or 180 Hz. There are several types of liquid crystal modulators that provide higher frame rates. An especially promising variant is the ferroelectric spatial light modulator (fSLM). Currently, they provide up to a 6 kHz frame rate [13] that can be a perspective for operative applications. A prerequisite for high-speed imaging using DMD and fSLM is the use of bi-grade (binary) images. Thus, holograms are recorded by a digital camera, binarized, displayed on DMD/fSLM, and optically reconstructed by incident radiation.

Binary computer-generated and digital holograms are used for various DMD applications: holographic displays [2,14,15,16], scattering media characterization [17], beam modulation [18], phase imaging [19], additive technologies [20], data coding [11], packaging [21], etc. Binary holograms are also widely used in other areas: image recognition and watermaking [22,23], creation of specially shaped beams [24], visualization of scanning holographic information [25], data compression [26] and storage [27], optical encryption [28], etc.

Many techniques have been investigated and developed for hologram binarization. The most common are local and global thresholding methods [2,22,29,30,31,32,33] and error diffusion techniques [15,34,35,36,37,38,39,40,41,42,43]. They are used both for binarization of optically registered digital holograms [15,30,32,33,37] and for computer-generated holograms [29,34,38,39,40,43]. The principle of thresholding is comparing the brightness values of each pixel in the image with some threshold. If the threshold value is higher than a pixel’s brightness value, that pixel is assigned a zero value, otherwise a maximum brightness value (“1”). The principle of binarization by error diffusion is to sequentially compare the brightness of image pixels to some threshold (usually half the maximum intensity). Then, the difference of these values (error values) with different weighting coefficients is distributed among the pixels that have not yet passed, adjacent to the pixel in question. There are other binarization methods, which are less frequently used and usually focus on computer-generated hologram processing: direct binary search algorithms [44,45,46], iterative techniques of kinoform generating [47,48], sampling [14,49], and others.

There are many implementations of digital holograms binarization based on thresholding and error diffusion. However, according to the results of the comparison of methods for optically registered digital holograms [15,32,33,42], the following was found. It is impossible to determine the universal method providing stable high quality for processing holograms of objects of different types: three-dimensional scenes, plane binary, and grayscale objects. It is only possible to reveal the groups of methods providing the best and the worst quality of image reconstruction for a particular type of objects. Accordingly, the obtained results should be used as a basis for binarization of selected digital holograms.

This paper proposes an adaptive binarization method based on a combination of local threshold processing, hologram block division and error diffusion procedure (the LDE method). The method takes into account features of hologram processing of different types of objects. Section 2 briefly describes different binarization methods: local and global thresholding, error diffusion and dot diffusion, and the developed LDE method. In Section 3, the effect of the method parameters on numerically and optically reconstructed images is analyzed. The quality is also compared with other binarization methods. The main results are given in the conclusions.

## 2. Methods for Binarizing Digital Holograms

### 2.1. Local and Global Thresholding

Threshold binarization is the most popular digital image binarization method [50]. It is widely used in processing textual images [51], unevenly illuminated documents [52], degraded archival [53] and historical [54] documents, etc. Threshold binarization has also become widely used in holography [2,22,29,30,31,32,33]. Algorithms are based on separation of background components from the object ones.

There are the two most common groups of threshold binarization [33] methods: global (Otsu [55], Kittler and Illingworth [56], Renyi [57] methods, etc.) and local thresholding (Niblack [58], Bernsen [59], Sauvola [60] methods, etc.). In the case of global threshold, a single threshold value is computed. It is constant for the entire image. This can lead to nulling of a significant number of information components of the hologram. In the case of local adaptive methods, the image is usually divided into the blocks [50]. For each block of pixels, a different threshold value is calculated. Such adaptivity of local methods allows for saving a higher percentage of informative components during binarization. Accordingly, a higher quality of image reconstruction is achieved. There are also iterative methods of global binarization by threshold [56,61]. They provide a higher quality of image reconstruction. However, the calculation time of binarization is long.

Thus, adaptive local methods are optimal from the point of view of both reconstruction quality and speed of processing. Therefore, they will be used in the development of a universal hologram binarization method.

### 2.2. Error Diffusion

Error diffusion is another popular method for binarizing digital images [62]. It is widely used for grayscale image rasterization in document processing, printing of monochrome images, etc. In addition, error diffusion based binarization has become widely used for holographic data processing [34,35,36,37,38,39,40,41,42,43].

Error diffusion techniques can be divided into two main groups [15]: standard (classical) (Floyd–Stenberg [63], Stucki [64], Atkinson [65] methods, etc.) and dot (Knuth [66], Arney, Anderson and Ganawan [67], Fung Chan [68] methods, etc.). Standard error diffusion techniques have a number of parameters: the values and number of weighting coefficients (weighting matrix; kernel of error diffusion), direction and order of bypass of pixels in the image, etc. In the case of dot diffusion, the error value is propagated to all eight surrounding pixels, including those already traversed [66,67,68].

It is also possible to use not only standard methods, but also modified error diffusion algorithms (e.g., using different image metrics as thresholds) for the binarization of digital holograms [15,69,70]. This could provide an additional quality improvement compared with the case of standard algorithms [15]. Thus, more comprehensive thresholding algorithms (e.g., adaptive local methods) in the error diffusion procedure should allow for improving results.

### 2.3. Developed Method of Hologram Binarization (the LDE Method)

Based on the analysis of implemented methods by threshold and error diffusion, a combined adaptive method for binarization of digital holograms has been developed. This method includes adaptive local determination of threshold for each block of hologram pixels. Then, an error diffusion procedure is carried out taking into account the calculated thresholds. As a result, the quality of hologram binarization is improved by joint use of different approaches. The algorithm for the developed LDE method consists of the following steps:

1. Division hologram into blocks of size S × S pixels;
2. Determination of adaptive local threshold for each pixel of each block;
3. Error diffusion part:
  - Consistently comparing the brightness value of each pixel with calculated threshold value;
  - Binarization the pixel value;
  - Determination of the corresponding error value;
  - Diffusion of the error value among neighboring considered pixels taking into account weighting coefficients and pixel bypass matrix;
4. Merging blocks into a single hologram.

**Figure 1:**
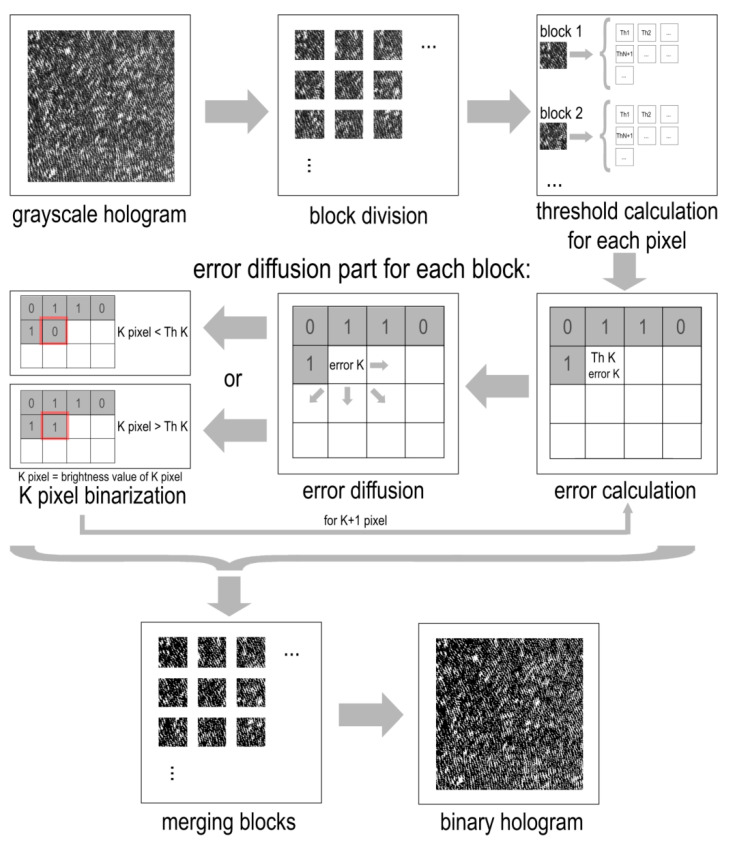
A schematic of the proposed LDE method.

A schematic of the method is shown in Figure 1.

The adaptive calculation of threshold for each local area improves the accuracy of determining more correct “0” and “1” pixel values. Distribution of error values among the pixels avoids the appearance of single brightness peaks after thresholding. Use of complex pixel bypass matrices avoids both random accumulation of error values and the resulting distortion of the interference fringes of the hologram. Thus, the algorithm combines the advantages of local thresholding methods and error diffusion techniques. It allows for varying the binarization parameters in accordance with the features of each specific hologram.

## 3. Results

### 3.1. Numerical Experiments

#### 3.1.1. Numerical Experiment Conditions

**Figure 2:**
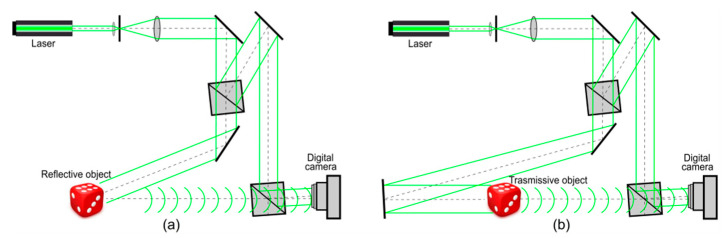
Off-axis digital holographic scheme in regimes of object’s illumination: “in reflection” (**a**) and “in transmission” (**b**).

In this work, experimentally recorded off-axis digital holograms of flat objects and 3D-scenes were used. The holograms were binarized by global and local thresholding methods, standard and dot error diffusion, and the proposed LDE method. Then, the object images were reconstructed, and the quality was assessed. The holograms were 2048 × 2048 pixels in size. The pixel size is 9 × 9 μm. The range of distances between objects and digital camera plane was 850 mm ÷ 1330 mm. The main elements of the off-axis digital holographic setup are given in Figure 2. Holograms of reflective (Figure 2a) and transmissive (Figure 2b) objects were used.

Images of flat objects or elements of a 3D-scene were reconstructed using the Fast Fourier Transform (FFT)-based Fresnel approximation method (single-FFT approach) [71,72]:

(1) $$R\left(x,y,z\right)=\frac{\exp\left(ikz\right)}{i\lambda z}\exp\left(\frac{i\pi \left(x^{2}+y^{2}\right)}{\lambda z}\right)\cdot FFT\left\{H\left(\xi ,\eta ,0\right)\exp\left(\frac{i\pi \left(\xi^{2}+\eta^{2}\right)}{\lambda z}\right)\right\},$$

where *FFT*{…}—the fast Fourier transform, *k*—wavenumber, *z*—the distance from the object element to the hologram, (*ξ*,*η*,0)—the coordinates in the hologram plane, (*x*,*y*,*z*)—the coordinates in the reconstruction plane, and *H*(*ξ*,*η*,0)—digital hologram.

**Figure 3:**
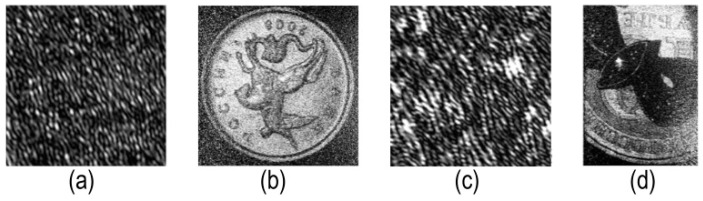
Fragments of digital holograms and corresponding reconstructed images: a coin (**a**,**b**) and a brooch with a coin (**c**,**d**).

Figure 3 shows fragments of examples of optically registered digital holograms and reconstructed images. Fragments of holograms are 128 × 128 pixels in size. The reconstructed image from the hologram shown in Figure 3a contains a coin (Figure 3b). Reconstructed from the other hologram (Figure 3c) contains a brooch with a coin (Figure 3d) [15,33,42]. These objects are reflective ones. The scene depths were 1 mm (Figure 3b) and 12 mm (Figure 3d). The distance from the objects to the hologram was 990 mm (Figure 3b) and from 1000 to 1012 mm (Figure 3d). Since the off-axis registering scheme was used, the object images are free of zero-order and twin image.

**Figure 4:**
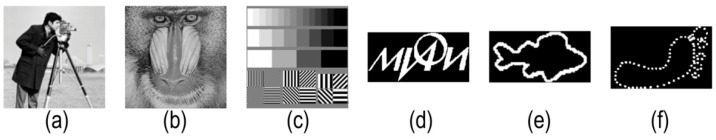
Examples of test grayscale (**a**–**c**) and binary (**d**–**f**) objects.

For verifying the LDE method on model holograms, additionally off-axis computer-generated digital holograms were used. Figure 4 shows the examples of test objects used in this work: grayscale (a–c) and binary (d–f) ones. They are used to synthesize digital holograms. The object images were positioned in the upper left corner of the black object field. This allows for preventing the reconstructed object image from overlapping with the zero-order and twin images. The phase is randomly distributed across the object field from 0 to 2π over all the pixels. Thus, the object is diffusely scattered. The range of distances between object and hologram plane was 300 mm ÷ 700 mm. Holograms were synthesized using the FFT-based Fresnel approximation method (single-FFT approach) [71,72]. The object wave was numerically propagated, then, the normally falling plane reference beam was added, the digital hologram synthesized, and an inverse FFT-based Fresnel approximation method (single-FFT approach) was used for the image reconstruction [73].

#### 3.1.2. Metrics for Assessing the Quality of Image Reconstruction

In the optical reconstruction of 3D-scenes from digital holograms, only the resulting intensity distribution is important for many applications, including, for example, 3D displays [2,15]. Therefore, to evaluate the quality of reconstruction, it was assumed that the information of interest lies only in the spatial intensity distribution of these objects. The values of the normalized standard deviation (NSTD) [74], peak signal-to-noise ratio (PSNR) [75], the structural similarity index (SSIM) [76], and correlation coefficient (CC) [52] were used as quality metrics for images reconstructed from binarized holograms. The NSTD value of the reconstructed object image relative to the original object image is determined by the following equation:

(2) $$\mathrm{NSTD}=\sqrt{1-\frac{{\left(\sum_{m=1}^{M}\sum_{n=1}^{N}A\left[m,n\right]\cdot B\left[m,n\right]\right)}^{2}}{\left(\sum_{m=1}^{M}\sum_{n=1}^{N}{\left(A\left[m,n\right]\right)}^{2}\right)\left(\sum_{m=1}^{M}\sum_{n=1}^{N}{\left(B\left[m,n\right]\right)}^{2}\right)}},$$

where *A*[*m*,*n*] and *B*[*m*,*n*] are the object images reconstructed from the original and binarized holograms, *M*,*N*—the quantity of rows and columns of pixels of the images, and *m*,*n*—the indexes of rows and columns. The NSTD value allows for determining the degree of difference between the images. If the NSTD value is 0, the images are identical, and, if the value is 1, they are completely different.

PSNR is another metric [75]. The PSNR value for two images *A*[*m*,*n*] and *B*[*m*,*n*] is calculated using the mean squared error (MSE):

(3) $$\mathrm{PSNR}=20\log_{10}\left(\frac{A_{max}}{\sqrt{\mathrm{MSE}}}\right),$$

(4) $$\mathrm{MSE}=\frac{1}{MN}\sum_{m=1}^{M}\sum_{n=1}^{N}{\left|A\left[m,n\right]-B\left[m,n\right]\right|}^{2},$$

where *A_max_* is the maximum signal value of reconstructed object image from the original digital hologram. For 8-bit images, *A_max_* = 255. PSNR allows for determining the degree of difference between images, when one image can be considered a noisy version of another. The higher the PSNR value, the greater the degree of the similarity of images.

SSIM [76] was also used as a measure of image quality. In a simple form, it is defined as follows:

(5) $$\mathrm{SSIM}=\frac{\left(2\mu_{A}\mu_{B}+c_{1}\right)\left(2\sigma_{A}\sigma_{B}+c_{2}\right)}{\left({\mu_{A}}^{2}+{\mu_{B}}^{2}+c_{1}\right)\left({\sigma_{A}}^{2}+{\sigma_{B}}^{2}+c_{2}\right)},$$

where *μ_A_*, *μ_B_*, *σ_A_*, *σ_B_*, and *σ_AB_* are the mean values, standard deviations, and cross-covariance for the images *A*[*m*,*n*] and *B*[*m*,*n*], and *c*_1_, *c*_2_ are constants. The range of SSIM values is between −1 and 1. A value of 1 is reached when the images are a perfect match.

CC is defined as follows [52]:

(6) $$\mathrm{CC}=\frac{\sum_{m=1}^{M}\sum_{n=1}^{N}\left(A\left[m,n\right]-\mu_{A}\right)\cdot \left(B\left[m,n\right]-\mu_{B}\right)}{\sqrt{\left(\sum_{m=1}^{M}\sum_{n=1}^{N}{\left(A\left[m,n\right]-\mu_{A}\right)}^{2}\right)\left(\sum_{m=1}^{M}\sum_{n=1}^{N}{\left(B\left[m,n\right]-\mu_{B}\right)}^{2}\right)}}.$$

If the CC value is equal to 1, the images are identical. The closer the CC value to 0, the lower the degree of similarity of images.

NSTD, PSNR, SSIM, and CC for almost flat objects were defined as a single value over object (for example, coin) area. NSTD, PSNR, SSIM, and CC for 3D-objects or 3D-scenes were defined as an average value over NSTDs/PSNRs/SSIMs/CCs for different focused 2D-elements.

#### 3.1.3. Effect of the LDE Method Parameters on the Reconstruction Quality

The developed LDE method has been tested on optically registered and computer-generated digital holograms. The local binarization method by the Otsu threshold [55] was used to determine local threshold. The Otsu thresholding had demonstrated one of the best results both in terms of quality and calculation speed [15,42]. The cases of division of digital holograms into blocks of different sizes: 4 × 4, 8 × 8, 16 × 16, 32 × 32 and 64 × 64 pixels were considered. Processing of larger blocks decreases the computation time and the accuracy of local determination of thresholds. Use of the smallest blocks (2 × 2) can have a negative effect on the interference pattern microstructure.

**Figure 5:**
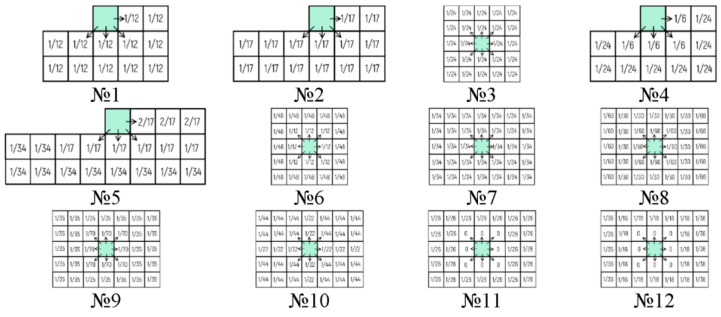
Weighting matrices of error diffusion for the proposed LDE method.

In [15], a number of results were demonstrated. For example, the highest quality of reconstruction was achieved using weighting matrices with a large number of coefficients (more than 10). Based on these results, 12 weighting matrices have been developed. They are shown in Figure 5.

**Figure 6:**
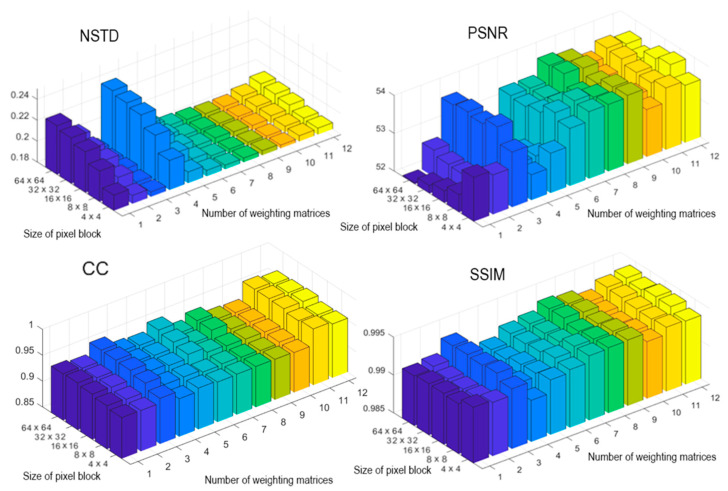
Quality metrics (NSTD, PSNR, SSIM, and CC) of image reconstruction from binarized holograms using the LDE method. The holograms were divided into blocks of different size. Twelve weighting matrices were used.

Optically registered holograms were binarized by the LDE method. Twelve mentioned weighting matrices were used. Figure 6 shows quality estimates (NSTD, PSNR, SSIM, and CC) of reconstructed images. The highest values for all metrics are achieved when the hologram is divided into the blocks of 8 × 8 and 4 × 4 pixels and weighting matrices №3, №8, and №9 are used. These blocks can be considered as the most appropriate ones for the LDE method.

**Figure 7:**
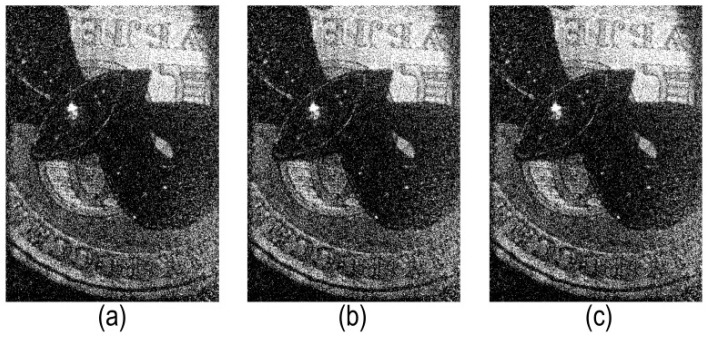
Examples of reconstructed images from holograms binarized by LDE method. Matrices №3 (**a**); №8 (**b**); and №9 (**c**) were used. The holograms were divided into blocks of 32 × 32 pixels.

Examples of reconstructed images from holograms binarized using the LDE method are shown in Figure 7. Three best matrices with hologram division into blocks of 32 × 32 pixels were used.

With these method parameters, the reconstruction quality improves over standard implementations of thresholding and error diffusion. The distance between the hologram and objects actually does not have an effect on the quality of the LDE method working. The type of object has more effect. The method provides especially significant improvement for simple objects’ hologram binarization. For example, for the optically recorded hologram shown in Figure 3c,d, the improvement was 11% and 18%, respectively. In the binarization of computer-generated digital holograms, quality was improved up to 30% compared with standard methods.

Dividing the hologram into blocks of pixels and calculating a threshold for each block allows for taking into account all local features of holograms. Such binarization preserves a significant part of the informative components. Thus, the advantage of the proposed method compared with standard implementations is adaptive, accounting for unevenly illuminated pixels and correlation of pixel brightness values. At the same time, unlike the best global thresholding methods (such as the iterative Kittler method [56]), the proposed method is not iterative. Accordingly, the reconstruction quality does not depend on the number of iterations and does not affect the computational time of the method. Due to the adaptivity of the threshold estimation in the error diffusion procedure, the LDE method can provide consistently high-quality reconstruction for different holograms with a minimal loss of original information.

#### 3.1.4. Comparison of Binarization Methods

The quality of image reconstruction from binarized holograms has been comparatively analyzed. Binarization methods based on five groups of algorithms were applied:

- Global thresholding (Kittler and Illingworth [56], Prewitt [77], Otsu [55], Renyi [50,57], and Shanbhag [78] methods);
- Local thresholding (Bernsen [59], Soille [79], Niblack [58], Sauvola [60], and local Otsu [55] methods);
- Standard error diffusion (Floyd–Stenberg [63], Jarvis [80], Stucki [64], Atkinson [65], and the simplest horizontal [81] methods);
- Dot diffusion (Knuth [66], Fung Chan [68], Liu [82], Arney, Anderson and Ganawan [67], and Guo [83] methods);
- Proposed adaptive LDE method (with weighting matrices №3, №8–9, №11–12, see Figure 5).

The algorithms of these methods are typical for each group and are based on different approaches. The choice of methods has been determined by [15,32,33,42]. For example, in [33], an analysis of local and global threshold methods for optically registered holograms is presented. It was found that the highest reconstruction quality is obtained using global Kittler [56] and Otsu [55] methods and local Otsu [55] and Niblack [58] methods.

**Figure 8:**
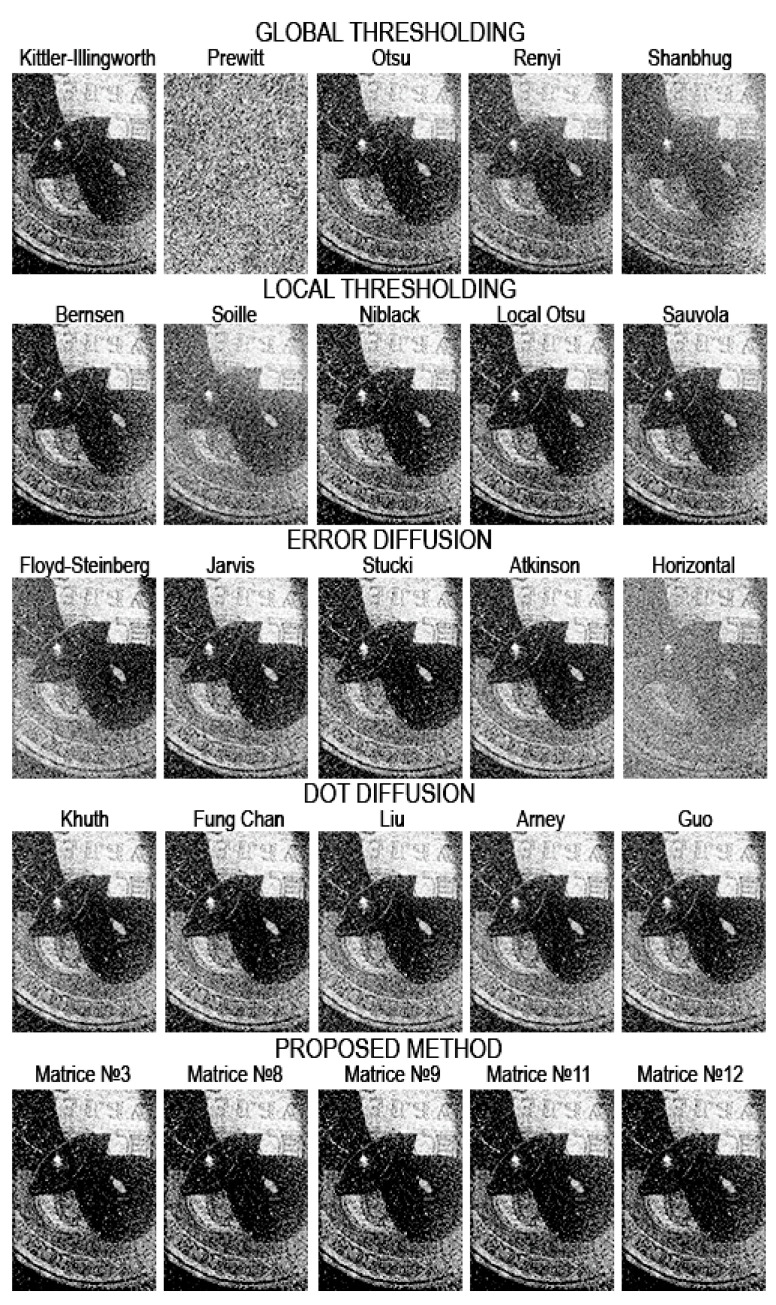
Examples of reconstructed images from binarized optically registered holograms. The original grayscale hologram is shown in Figure 3c,d.

**Figure 9:**
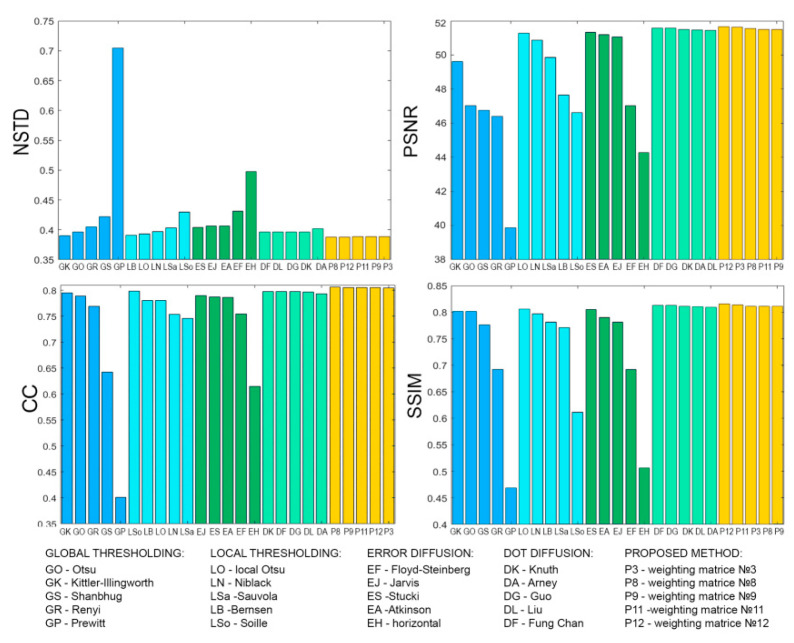
NSTD, PSNR, SSIM, and CC values for the reconstructed object images shown in Figure 8.

**Figure 10:**
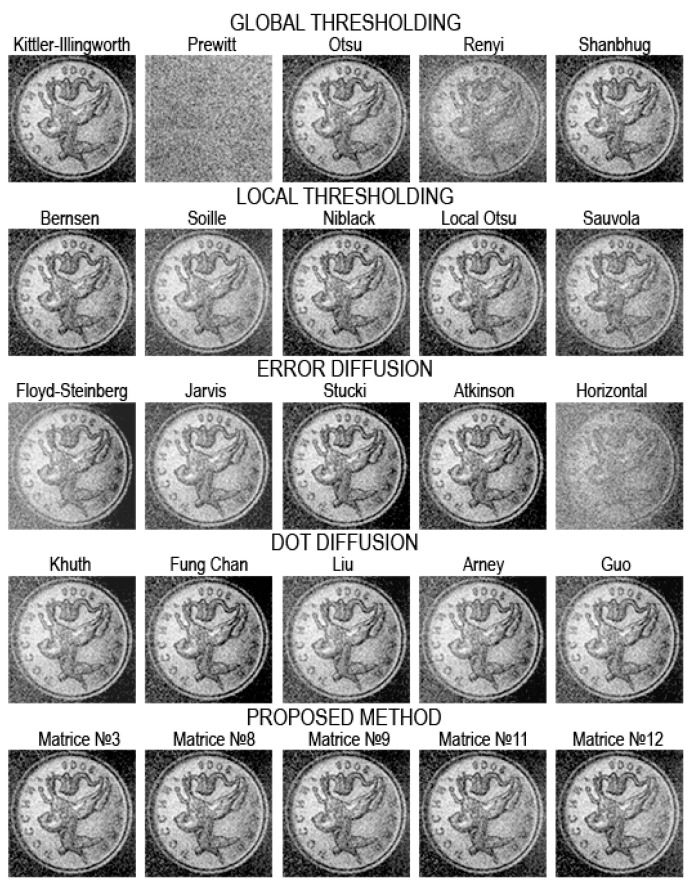
Examples of reconstructed images from binarized optically registered holograms. The original grayscale hologram is shown in Figure 3a,b.

**Figure 11:**
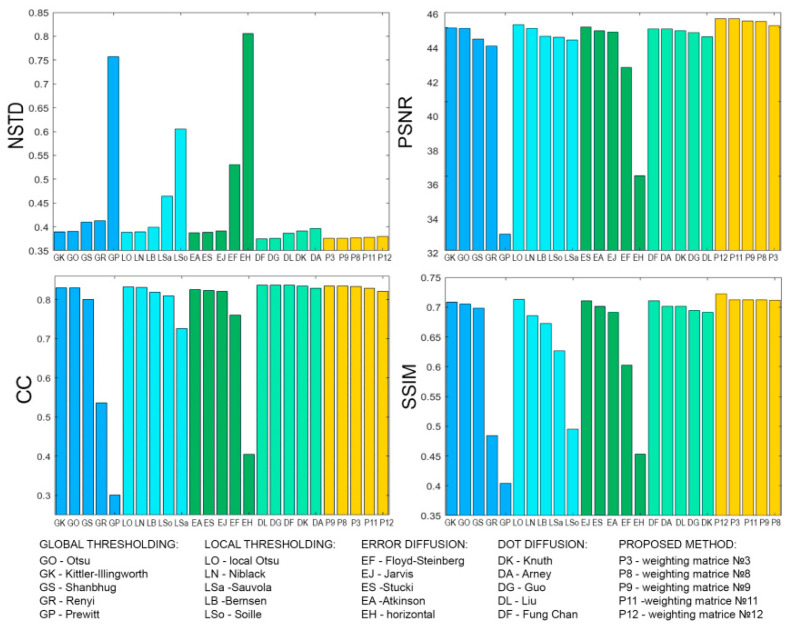
NSTD, PSNR, SSIM, and CC values for the reconstructed object images shown in Figure 10.

**Figure 12:**
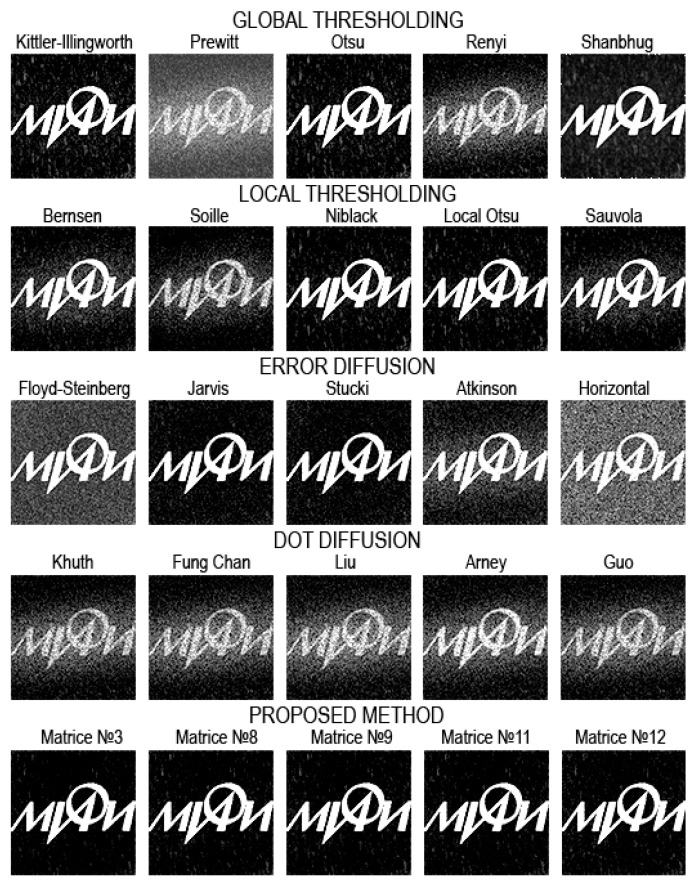
Examples of reconstructed images from binarized synthesized digital holograms. The original binary object image is shown in Figure 4d.

**Figure 13:**
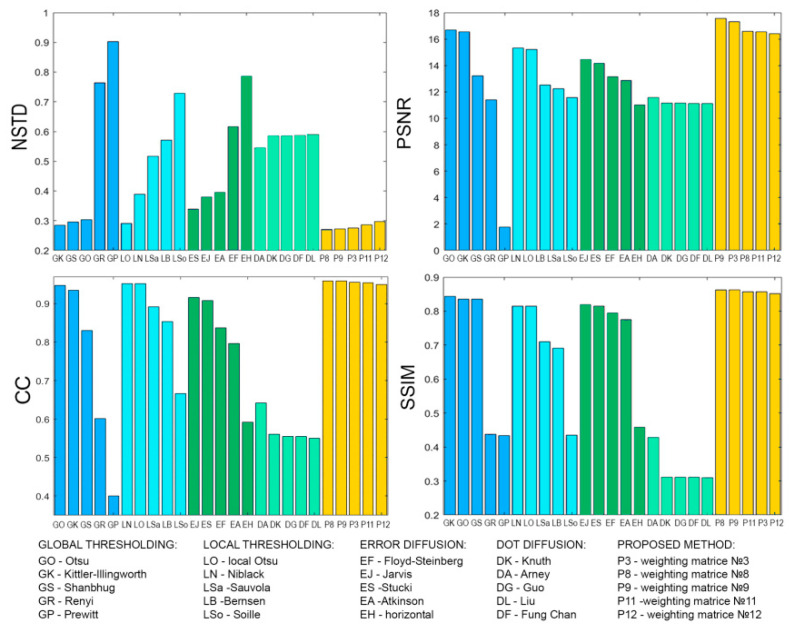
NSTD, PSNR, SSIM, and CC values for the reconstructed object images shown in Figure 12.

**Figure 14:**
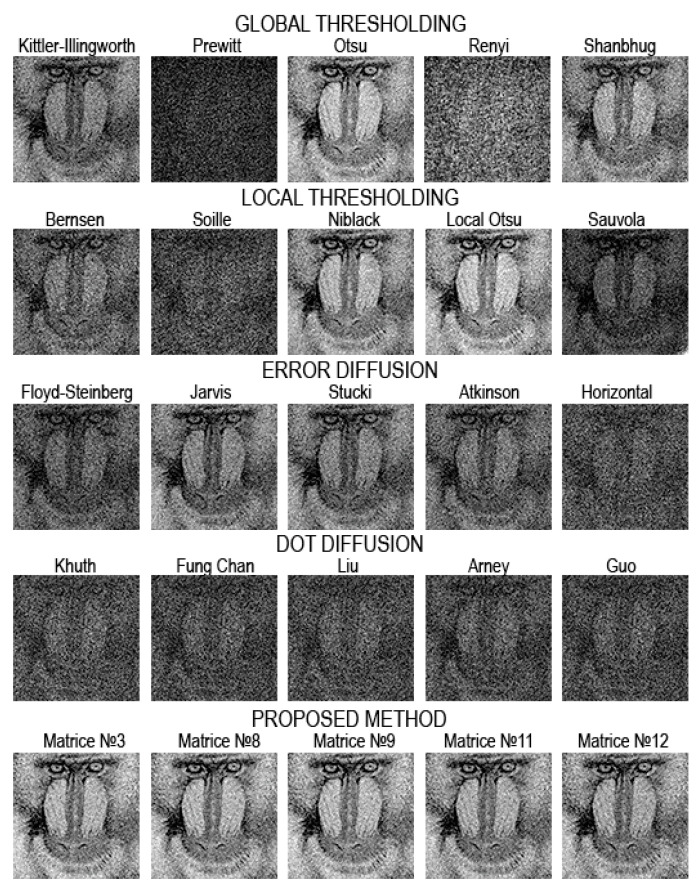
Examples of reconstructed images from binarized synthesized digital holograms. The original grayscale object image is shown in Figure 4b.

**Figure 15:**
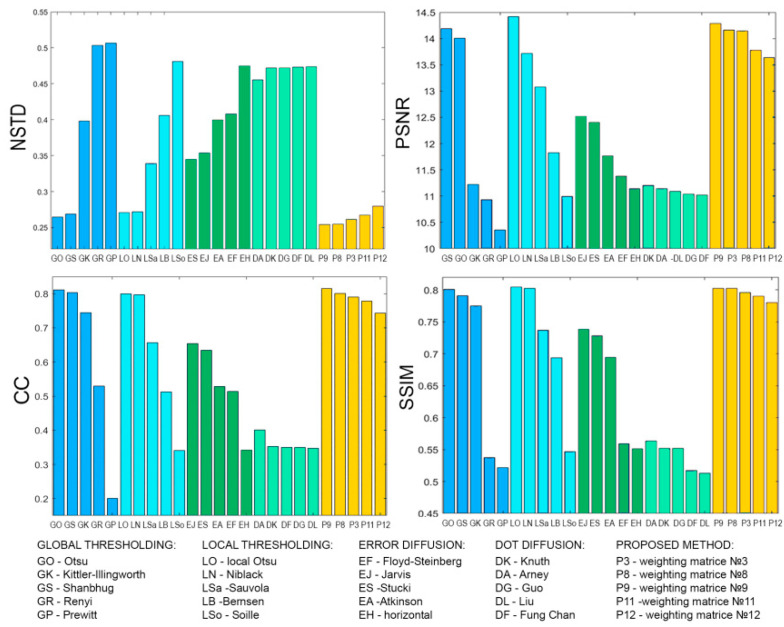
NSTD, PSNR, SSIM, and CC values for the reconstructed object images shown in Figure 14.

Figure 8, Figure 9, Figure 10, Figure 11, Figure 12, Figure 13, Figure 14 and Figure 15 demonstrate several results. Figure 8, Figure 10, Figure 12 and Figure 14 show examples of numerically reconstructed images from holograms binarized using the above methods. The corresponding values of quality metrics are shown in Figure 9, Figure 11, Figure 13 and Figure 15. As can be seen, the highest reconstruction quality is achieved using the proposed method as well as the Otsu, Shanbhag and Stucki methods. The worst reconstruction quality was obtained using global threshold binarization by Prewitt and Renyi methods, and local threshold binarization by Soille.

Figure 10 and Figure 12 show quality metrics for 3D-scene reconstruction from binarized optically registered holograms. The LDE method gives the best result. In addition, dot diffusion methods, the iterative global Kittler threshold, local and global Otsu method, local Niblack method, and Stucki error diffusion show good results. For the cases of binary (Figure 12 and Figure 13) and grayscale (Figure 14 and Figure 15) image reconstruction from binarized computer-generated digital holograms, the LDE method also showed the best results. Stucki’s standard error diffusion and thresholding local and global Otsu processing methods showed good results.

The results of the comparison confirm calculations for threshold and error diffusion techniques presented in [15,33]. In addition, the results in [15,33] demonstrate that the reconstruction quality can vary depending on the type of holograms and used objects. However, the reconstruction quality for different holograms remains consistently high for the LDE method.

Computation time of binarization was estimated. For example, let us use the MATLAB programming language and a laptop with an Intel Core i5-5200U CPU 2.2 GHz 4 GB RAM processor. The hologram size was 1024 × 1024 pixels. Average computation times were: 0.02 s for global non-iterative thresholding, 0.05 s for error diffusion techniques, 0.09 s for local thresholding, 0.1 s for the LDE method, and 8.5 s for global iterative thresholding. Computation time was specially estimated using a slow enough office laptop. It demonstrates wide possibilities of computation time increase. The computation times can be easily decreased a dozen times by using a high-speed computer only. The use of software algorithms can additionally speed up this process significantly [84].

Holograms are divided into square blocks in the LDE method. However, blocks can have other similar shapes. For example, they can be a rectangular one with size *S*_1_ × *S*_2_. Results of binarization are similar to those for square blocks. However, calculation for square blocks is simpler and faster. If *S*_1_ and *S*_2_ are very different, the results are changing relative to the square case and becoming worse. This is due to the nonlinear effect of such block size on interference fringes of the hologram.

Thus, the LDE method provides the highest quality for both optically registered and computer-generated digital holograms. In addition, it is the most universal among considered methods of binarization for different types of objects: for volumetric scenes, as well as for grayscale and binary objects.

### 3.2. Optical Experiments

Optical experiments have been performed to verify numerical estimates. Holograms were binarized by the global and local threshold, standard and dot error diffusion, and LDE method, and then reconstructed. Computer-generated and optically registered digital holograms of flat objects and three-dimensional scenes were used (examples are given in Figure 3 and Figure 4).

**Figure 16:**
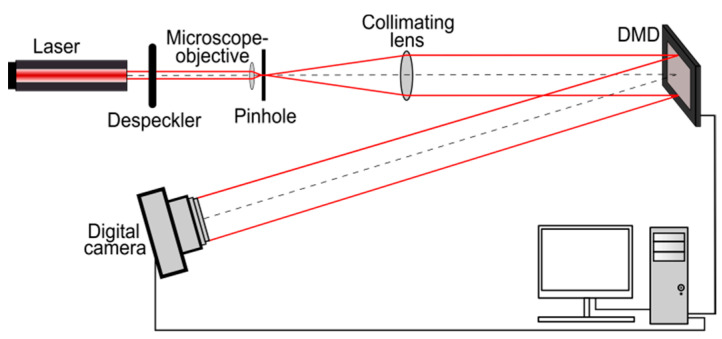
Schematic of optical hologram image reconstruction using DMD.

A schematic diagram of the experimental setup is shown in Figure 16. An He-Ne laser (633 nm, 10 mW) was used as the radiation source. To reduce speckle noise, an Optotune LSR-3010 despeckler was used. A pinhole was used for spatial filtering of the light beam. The obtained broadened beam illuminated a DMD DLP9500BFLN (1920 × 1080 pixels, frame rate up to 23 kHz, pixel size 10.8 × 10.8 µm). Binarized digital holograms were displayed on the DMD. A mirrorless digital camera Canon EOS M100 (CMOS sensor, 6024 × 4020 pixels, pixel size 3.7 × 3.7 µm) recorded the reconstructed images. The display of the holograms on the DMD, camera control, storage of the reconstructed images, and their processing were performed by a computer. The holograms were 256 × 256 to 1920 × 1080 pixels in size.

**Figure 17:**
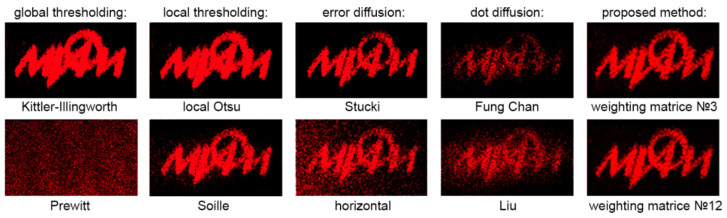
Examples of optically reconstructed images from computer-generated digital holograms. Results for best and worst methods of five groups of binarization methods are presented.

Figure 17 shows reconstructions from binarized computer-generated digital holograms of a binary object. For each of the five groups of methods, the best and worst reconstruction quality results are presented. The highest quality is achieved using the developed LDE method. In addition, high quality is obtained using Stucki’s method, which is the best among standard diffusion error techniques according to numerical experiments. Weighting matrices №3 and №12 are used for the LDE method. As can be seen, these parameters provide the best results for both numerical simulations and experimental optical reconstructions. The local binarization methods by threshold (best Otsu and worst Soille) and the global binarization method by Kittler threshold (best) also provide relatively high quality: objects are recognizable and fine details are preserved. The dot diffusion as well as in numerical reconstruction showed not very good results for computer-generated digital holograms where insufficient inhomogeneity of the hologram plays the major role. This result can be compensated by using as a threshold value in the error diffusion procedure more complex metrics instead of the standard value of half of the maximum intensity (127.5 digital units for 8-bit hologram file) [15]. The worst result was obtained for the global Prewitt’s thresholding. This method also showed worse results in numerical simulations, but, in optical experiments, the images become completely indistinguishable.

**Figure 18:**
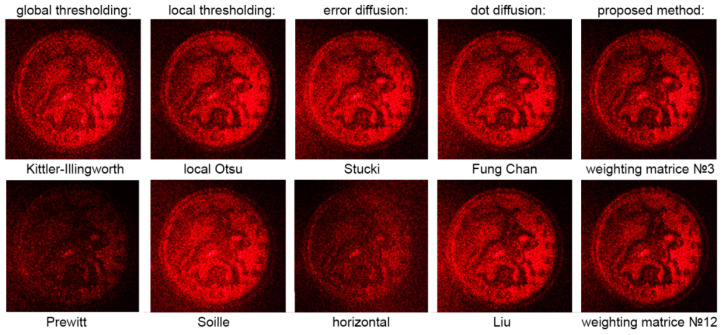
Examples of optically reconstructed images from registered digital holograms. Results for best and worst methods of five groups of binarization methods are presented.

Figure 18 shows examples of reconstructed images from binarized optically registered holograms. The cases of best and worst methods from each group are shown. As can be seen, even in the case of the worst binarization methods (Prewitt’s global thresholding and horizontal standard error diffusion), the object image remains but is barely recognizable. Dot diffusion provides much higher quality than in the case of binarization of computer-generated digital holograms. As stated earlier, this is due to non-uniformity of holograms and to the value of the threshold in the error diffusion procedure.

The quality of optically reconstructed images have been evaluated. The results of metrics averaging for all holograms (optically registered and computer-generated digital holograms) showed that the highest quality was obtained using the LDE method with matrix №12. In addition, high quality was obtained for Stucki’s standard diffusion error and Otsu’s local threshold. Relative to the standard global Otsu threshold, the reconstruction quality is higher on average by 22% for the LDE method. Relative to the standard Floyd–Stenberg error diffusion, the quality is higher on average by 19% for the LDE method.

For optically recorded holograms only, the reconstruction quality for the LDE method is on average 18% higher than that of standard methods. The use of Fung Chang’s dot diffusion method achieves an improvement in reconstruction quality compared to standard methods. However, for computer-generated digital holograms, the other dot diffusion methods show results achievable with standard methods.

**Figure 19:**
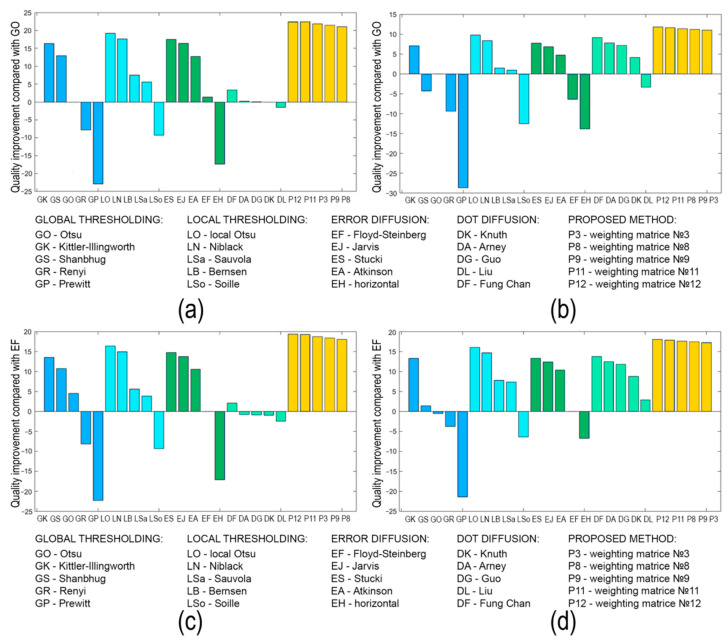
Values of quality metrics enhancement (NSTD, PSNR, SSIM, CC) of reconstructed images from holograms binarized by different methods relative to the global Otsu threshold (GO: (**a**,**b**)) and Floyd–Steinberg error diffusion (EF: (**c**,**d**)). Cases of using all holograms (**a**,**c**) and only optically registered holograms (**b**,**d**) are given.

These results and the results for the other methods are shown in Figure 19. The relative increase (in %) in the quality metrics of optical image reconstruction from binarized holograms is shown. A comparison is performed relatively of global Otsu thresholding (Figure 19a,b) and the standard Floyd–Steinberg error diffusion technique (Figure 19c,d). An average of four metrics for all used holograms (both computer-generated and optically recorded digital holograms; Figure 19a,c) and only for recorded digital holograms (Figure 19b,d) are given. The optical reconstruction results confirm the results of the numerical simulations presented in Section 3.1.3 and Section 3.1.4.

Thus, the LDE method is the most universal. Stucki’s error diffusion technique and local Otsu thresholding also give good results. The LDE method is suitable for processing different types of holograms. Additionally, it has a quite simple algorithm. In addition, the method is non-iterative, which allows high speed hologram binarization. One of the possible modifications of the method is iterative search [36,37] (e.g., search for the best weighting matrix used in the error diffusion procedure). This modification would allow for achieving a more accurate accounting of pixel brightness values of the original hologram. However, iterativity should increase the computation time of the method in multiples of the number of iterations. The use of software algorithms can significantly speed up this process [84]: for example, graphic processing units [85], field-programmable gate arrays [86], etc. It would also be necessary to determine the optimum conditions to achieve an increase in reconstruction quality in a minimum number of iterations.

The proposed method enables better hologram binarization actually without increasing the computational times of the procedure. This is particularly useful for high-speed optical reconstruction of three-dimensional scenes.

## 4. Conclusions

The paper considers the subject of binarization of digital holograms and proposes a method. The method uses a combination of local thresholding, block division, and error diffusion procedure. Thirty-two binarization methods were compared: 5 local and 5 global thresholding, 5 standard and 5 dot error diffusion, and 12 implementations of the proposed method. Off-axis optically registered and computer-generated digital holograms were binarized and analyzed. Optimal parameters of the proposed method providing the highest quality of reconstruction were determined.

The best results are provided when using the proposed method with weighting matrices with a large number of coefficients (for example, matrix №12, see Figure 5). The method is compared with others in numerical simulation and in real optical experiments using a binary DMD. The reconstruction quality for the proposed method is on average 22% and 19% higher in comparison to standard threshold binarization and error diffusion techniques, respectively. The optical reconstruction results confirm the numerical simulation ones.

Possible improvement is iterative search for more accurate accounting of the pixel brightness. In addition, more correct choice of the size of hologram blocks can improve the binarization quality. However, this should significantly decrease the method speed. It would also be necessary to determine the optimum conditions for minimization of iteration number.

The results can be useful in the binarization of digital holograms for both processing and storage tasks, as well as for high-speed reconstruction and beam shaping using DMD and fSLM.

## Data Availability

The data that support the findings of this study are available from the corresponding author, P.A.C., upon reasonable request.
